# Investigating Gene Function for Neuronal Survival After Metabolic Stress Using Semi-Automated Fluorescence Microscopy and Automated Image Analysis

**DOI:** 10.3389/fnmol.2018.00393

**Published:** 2018-11-02

**Authors:** Kristin Wendland, Andreas Meisel, Philipp Mergenthaler

**Affiliations:** ^1^Charité—Universitätsmedizin Berlin, Department of Experimental Neurology, Berlin, Germany; ^2^Charité—Universitätsmedizin Berlin, NeuroCure Clinical Research Center, Berlin, Germany; ^3^Charité—Universitätsmedizin Berlin, Center for Stroke Research Berlin, Berlin, Germany; ^4^Charité—Universitätsmedizin Berlin, Department of Neurology, Berlin, Germany; ^5^Berlin Institute of Health (BIH), Berlin, Germany

**Keywords:** neuronal survival, cell-based assay, transfection, semi-automated microscopy, automated image analysis

## Abstract

Overexpression approaches and fluorescence microscopy techniques allow investigating important spatiotemporal aspects of gene regulation as well as quantifying gene function. Consequently, fluorescence microscopy techniques help answer important questions on gene regulation such as addressing the role of a specific gene product for neuronal survival under different treatments. Here, we describe a versatile tool to measure effects of a transfected gene of interest on neuronal survival upon metabolic stress. We focus on nutrient starvation of cultured rodent primary neurons as a model of metabolic stress but our approach can easily be generalized and adapted to other cell types or to investigate single gene function in regulating neuronal survival under various conditions.

## Introduction

The regulation of gene expression provides a critical link between the genome and the phenotype of a cell in general and for neuronal function and survival in particular. However, understanding this link in all its complexity has not yet been accomplished. Especially transcriptomic and proteomic methods are used to unravel this elaborate and multi-layered system of interactions between genes, RNA molecules and proteins. However, important spatiotemporal information is lost when genetic, epigenetic or proteomic approaches are used as the sole determinant of gene function. In contrast, the combination of overexpression approaches and fluorescence microscopy techniques allows addressing the important “where and when” aspects of gene regulation as well as quantifying gene function. Indeed, fluorescence microscopy techniques help answer important questions on gene function such as addressing the role of a specific gene product for neuronal survival under different treatments. As such, fully automated microscopes enable the acquisition of the large number of images needed to quantitatively analyze these important gene expression questions (Muzzey and van Oudenaarden, 2009).

CellProfiler is an easy to use open-source image-analysis software, that enables the generation of image processing pipelines to measure and quantify biological phenotypes automatically from thousands of images (Carpenter et al., 2006; Carpenter, 2007; Kamentsky et al., 2011). It provides image segmentation and measurement algorithms as modules that can easily be arranged in an image-analysis pipeline to identify and quantify biological features and objects. An advantage of this technology is that these modular pipelines can easily be adapted and shared with colleagues. Various biological features of different cell types including yeast, mouse, rat and human cells have been identified using CellProfiler software (Bray et al., 2015; Gasparini et al., 2017).

The B-cell lymphoma-2 (Bcl-2) protein family consists of pro- and anti-apoptotic master regulators of cell death (Fricker et al., 2018; Kale et al., 2018). Bcl-extra large (Bcl-XL) is a prototypic anti-apoptotic member of this family. Apart from the role of the Bcl-2 family in brain development (Fricker et al., 2018), ample evidence link the Bcl-2 family members to a variety of central nervous system pathologies including stroke and excitotoxic injury (Engel et al., 2011; D’Orsi et al., 2012; Niu et al., 2017) and demonstrate strong neuroprotective function of Bcl-XL (González-García et al., 1995).

Here, we provide a general approach for using semi-automated image acquisition and automated image analysis to investigate the effects of a transgene of interest on neuronal survival under nutrient deprivation. Primary embryonic rodent cortical neurons are cotransfected with a nuclear marker (H2B-GFP) and Bcl-XL containing vector each as a positive control (PC) or an empty vector as a negative control (NC). In principle, plasmid vectors expressing the gene of interest could be cotransfected with H2B-GFB to investigate its function compared to the PCs and NCs. After 10 days in culture, images of H2B-GFP-expressing neuronal nuclei are captured employing semi-automated fluorescence microscopy and subsequently analyzed using CellProfiler to quantify viable neurons by automatically identifying and quantifying H2B-GFP expressing nuclei. Ultimately, the number of counted nuclei after treatment divided by the number of nuclei counted before treatment provides a measure for the anti- or proapoptotic effect of a gene of interest.

## Materials and Methods

### Preparation of Primary Cortical Neurons

Wistar rats or C57bl/6j mice were handled in accordance with institutional guidelines and regulations, and with permission of the *Landesamt für Gesundheit und Soziales (LAGeSo), Berlin*. Brains of day 17 rat embryos (E17) or day 15 old mouse embryos (E15) were isolated and cortices were dissected as described (Mergenthaler et al., 2012). Note that mating time needs to be short (4–6 h) to ensure exact age of embryos as this significantly affects transfection efficiency. Furthermore, hippocampus and olfactory bulb should be removed from cortices to generate homogeneous cortical cultures.

### Plasmids and Cloning

The Ubiquitin promoter sequence from pLVUT-tTR-KRAB (Szulc et al., 2006) was inserted into the promotorless vector pd2EGFP-N1 (Clontech, Takara Bio Europe, St-Germain-en-Laye, France) using PCR to create plasmid p1-Ubi-BclXl which served as PC. Plasmid pVitro2-neo-mcs (InvivoGen, San Diego, CA, USA) served as NC. Generation of pCAG-H2B-eGFP has been described (Mergenthaler et al., 2012). pLVUT-tTR-KRAB was a gift from Patrick Aebischer and Didier Trono (Addgene plasmid # 11651).

### Transfection and Cultivation of Primary Rat Cortex Neurons

Transfection of primary cortical neurons has been described (Mergenthaler et al., 2012). Briefly, 3 × 10^6^ neurons were combined with 5 μg DNA and 100 μl electroporation buffer (EB, 15 mM MgCl_2_, 192 mM NaCl, 9.6 mM KCl) and transfected using a Nucleofector-II (Amaxa, Lonza) device using program O-003 for rat neurons or O-005 for mouse neurons. Immediately after transfection, neurons were resuspended in 500 μl DMEM (Biochrom) supplemented with 3.7 g/l NaHCO_3_, 4.5 g/l d-glucose, 10% FCS. Next, 4 × 10^4^ neurons per well (~2.6 × 10^5^ cells/cm^2^) were plated in poly-D-lysine (PDL, Sigma, final concentration 20 μg/ml) and collagen pre-coated half area 96-well imaging plates (Greiner μClear, #675090). Medium was changed 4 h after transfection to remove dead cells and debris. Twenty-four hours after transfection cells were gently washed with 1× PBS and Neurobasal-A (NBM-A, Thermo Fisher Scientific, 2 mM L-glutamine) supplemented with B27 (Thermo Fisher Scientific) was added. One week after transfection, 50 μl of culture medium was removed and replaced with fresh NBM-A + B27. Cells were cultivated for 10 days. Note that the desired cell density may differ depending on the experimental setting and may need to be determined empirically. The optimal cell number for plating and therefore cell density depends on the number of dead cells accumulating during preparation. Moreover, electroporation itself influences the viability of neurons. 50%–70% of neurons die within 12 h after electroporation but the remaining cells grow well in culture. Cotransfection rate was approximately 90% as previously described (Mergenthaler et al., 2014).

### Semi-Automated Microscopy

On day 9 after transfection, culture medium was collected in 96-well plates and rat primary neuronal cultures were washed once with PBS. Cells were imaged in Live Cell Imaging Solution (LCIS, Thermo Fisher Scientific) containing 140 mM NaCL, 2.5 mM KCl, 1.8 mM CaCl_2_, 1.0 mM MgCl_2_, 20 mM HEPES, pH 7.4. Semi-automated image acquisition was performed using a fully automated Leica DMI 6000 microscope equipped with a Leica HCX PL FL L 20×/0.4 objective and a Leica DFC360 camera. The microscope was controlled by the Leica LAS AF v. 2.7 software with HCS A Matrix screener extension software. However, any automated research-grade inverted fluorescence microscope can be used for this type of assay, provided the controller software allows storing x-y coordinates to image the same fields of view (FOV) at subsequent time points. Using a 96-well template, 16 images per well from 10 wells per transfection were collected, adding up to a total of 160 images per condition. Likewise, on day 10 after transfection culture medium was removed, cells were washed once with 1× PBS and the same FOV were imaged 24 h after treatment. Images of transfected mouse cultures were used in Figure 2 and Supplementary Figures S1, S2, for illustration purposes.

**Figure 1:**
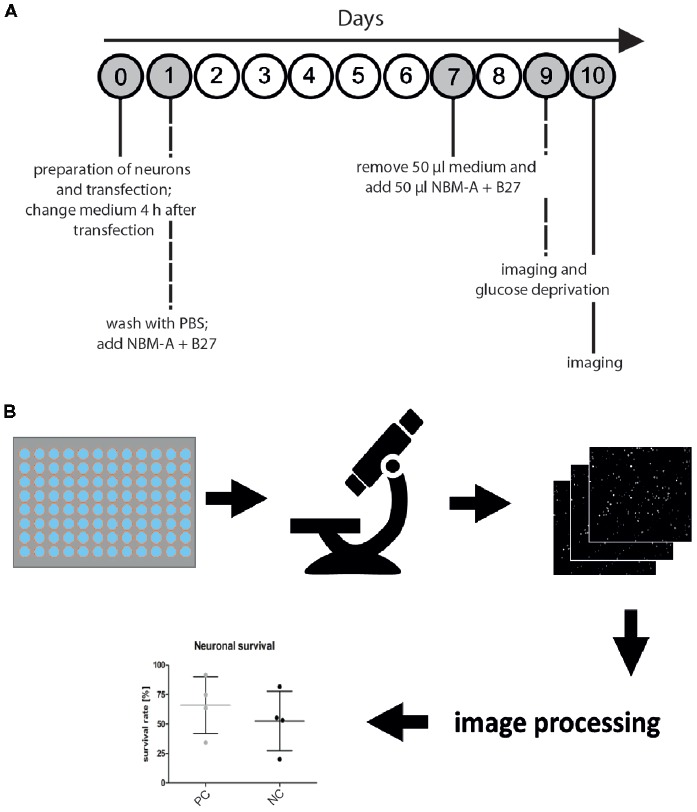
Microscopy-based neuronal survival assay. **(A)** General workflow. Overview of the timing for cell culture and imaging to measure the functional effects of a gene of interest on neuronal survival. First, primary rat cortical neurons are prepared from rat embryo brains and subsequently cotransfected using electroporation with a nuclear marker (i.e., H2B-GFP) and a gene of interest. To remove cellular debris due to detrimental effects of electroporation, medium is changed 4 h after transfection. On day 1 after transfection, another washing step and medium replacement are performed, followed by a feeding step on day 7 after transfection. On day 9 after transfection, images are acquired (semi-automated image acquisition) before glucose deprivation (GD) and 24 h after treatment (day 10 after transfection). **(B)** Seeding of transfected primary rat cortical neurons in 96-well imaging plates is followed by semi-automated microscopy with an acquisition of 16 images per well of neurons transfected with the nuclear marker H2B-GFP. Subsequently, automated image analysis is performed in CellProfiler using a custom image processing pipeline. Fluorescent nuclei are identified as primary objects and quantified for each image. Ultimately, neuronal survival is compared to experimental positive (positive control, PC) and negative controls (NC).

**Figure 2:**
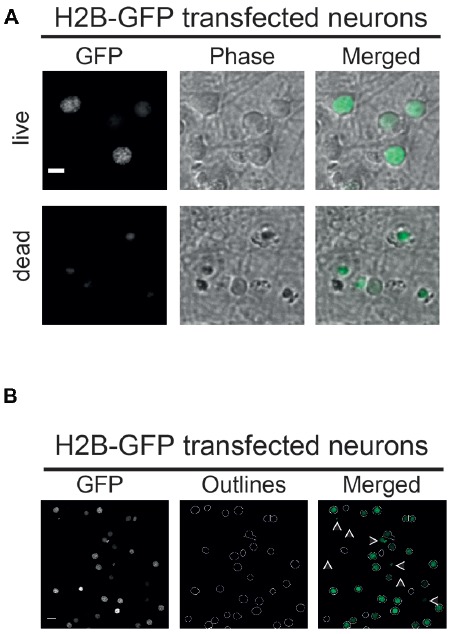
**(A)** Vital and non-vital neuronal nuclei. H2B-GFP transfected neurons displaying different morphological features depending on their condition. Nuclei of dead neurons (lower panel) are smaller in size and less structured compared to nuclei of live neurons (upper panel). Phase: phase contrast; scale bar: 10 μm. **(B)** Parameter-dependent detection of H2B-GFP transfected nuclei. Using a defined pixel diameter and a fluorescence intensity threshold, only vital neuronal cells are detected using the CellProfiler pipeline. Neurons with low expression of H2B-GFP amounts are excluded by the pipeline as well as nuclei that are too small in size, as indicated by white arrows; scale bar: 10 μm.

### Metabolic Deprivation of Primary Rat Cortex Neurons

After imaging of neuronal cultures on day 9 after transfection, cells were subjected to glucose deprivation (GD) for 6 h at 37°C and 5% CO_2_ as previously described (Sunwoldt et al., 2017) but using LCIS for incubation. LCIS was removed after imaging and replaced with the collected culture medium. Cells were maintained at 37°C, 5% CO_2_. Control cells did not undergo GD, but collected culture medium was added directly after imaging. See Figure 1 for a timeline of the entire workflow. In principle, for metabolic deprivation of neurons, oxygen-glucose deprivation (OGD), oxygen deprivation (OD), or GD can be performed. For a description of OGD and OD see (Mergenthaler et al., 2014).

### Automated Image Analysis Using Cellprofiler

CellProfiler 2.2.0 was downloaded from the CellProfiler webpage^1^. We set up the image-processing pipeline as follows: after loading the images, primary objects are directly identified. Preliminary experiments demonstrated that morphing and illumination correction was not necessary in our case since the signal to noise ratio of our images was sufficiently high. Then, fluorescent nuclei were counted based on two parameters: pixel diameter and by thresholding. The pixel diameter allows the pipeline to make a distinction based on size. Especially when using this pipeline for image analysis after GD, live and dead nuclei must be clearly separable. To empirically determine the pixel diameters, various live and dead nuclei (determined by morphology) were manually measured using the “Measure length” tool provided by CellProfiler. Therefore, the pixel diameter for live nuclei was set to 23–40 pixels, to exclude dead cells from the analysis. Figure 2 illustrates the differences in size between live and dead neuronal nuclei. Note that nuclei touching the image border are automatically excluded from analysis. The thresholding method affects whether a given pixel is recognized as foreground or background. A higher threshold value will favor the brighter regions of an image, whereas a low threshold value will include dim region of the image. CellProfiler can either automatically calculate the threshold from a choice of different thresholding methods (e.g., Otsu, Mixture of Gaussian (MoG), Background, Robust Background), or the user can manually enter a value between 0 and 1 defining the threshold. Here, we used MoG as the thresholding method. The MoG algorithm assumes that each pixel belongs to either foreground or background class and is recommended if less than 50% of the image is foreground. As the last module, all identified objects as well as other parameters including fluorescence intensity values for each nucleus were exported to a CSV file. In addition, as a quality control step, the pipeline outlines the detected nuclei and saves them as images. See Supplementary Note 1 for details.

### Calculation of Cell Death Parameters

Neuronal nuclei are scored as live or dead depending on the parameters mentioned above. Next, the total number of “live” nuclei after GD was divided by the number of live nuclei before GD and the percentage plotted and statistically analyzed.

### Statistics

Data were analyzed and plotted in GraphPad Prism. Details are indicated in the figure legend.

### Notes/Troubleshooting

Counting fluorescent nuclei is a straightforward phenotype. Various tools, including overexpression of fluorescent cellular markers, (fusion proteins, e.g., nuclear target sequence fused to GFP), immunofluorescence staining of transcriptions factors or counterstains (e.g., 4′,6-diamidino-2-phenylindole, DAPI), are available to sufficiently stain nuclei. However, detecting nuclei can be challenging due to segmentation problems caused by clumped nuclei. Therefore, cell density observation and evaluation are crucial to obtain meaningful nuclei counts.

## Objectives and Validation

The neuronal survival assay described herein implements different experimental procedures (Figure 1). For optimal performance of the assay, primary rat cortical neurons are seeded in 96-well imaging plates after transfection. On day 9 after transfection, semi-automated image acquisition using a research-grade fluorescence microscope is performed with subsequent nutrient starvation (i.e., glucose starvation) of neuronal cultures. In addition, images are acquired 24 h after GD with the objective to capture cells that survived or died. Next, images displaying H2B-GFP expressing neuronal nuclei are analyzed using the CellProfiler pipeline described above. Among other parameters, detected nuclei are outlined and saved as images. This step serves as quality control to detect potential errors in image analysis. Finally, quantitative analysis of the obtained data (i.e., nuclei count) enables a direct evaluation of a beneficial or detrimental effect of the investigated genes of interests. Here, the prototypic neuroprotective and anti-apoptotic gene Bcl-XL was investigated.

The functionality and applicability of the developed microscopy-based neuronal survival assay and subsequent image analysis using CellProfiler software is best illustrated using a simple experimental set-up. Thus, here we will only focus on a PC- and NC-transfected neurons subjected to GD as an example of metabolic stress. For this, a Bcl-XL containing plasmid was cotransfected with a nuclear marker (H2B-GFP) as the PC or an empty vector as the NC. The strong anti-apoptotic and neuroprotective function of Bcl-XL has been widely recognized and therefore constitutes a suitable PC to measure neuronal survival or cell death in similar assays (Youle and Strasser, 2008; Mergenthaler et al., 2012; Kale et al., 2018). Consequently, either pro- or anti-survival effects of the investigated gene of interest compared to the PC and NC can be measured using this assay. As described in further detail in the “Materials and Methods” section, images of H2B-overexpressing nuclei are collected. Subsequently, images are analyzed using a CellProfiler pipeline. Figure 2 illustrates the morphologies of H2B-GFP expressing neuronal nuclei (Figure 2A), the outlined nuclei detected after analysis with the CellProfiler pipeline and a merged image (Figure 2B). Furthermore, low resolution images of stained (Hoechst) and transfected (H2B-GFP) neurons show a general overview of the cultures (Supplementary Figure S1). The pipeline detects neuronal nuclei based on two user-defined parameters: pixel diameter and fluorescence intensity threshold. Note that, nuclei falling below a defined intensity or size threshold are excluded by the software, as indicated by white arrows (Figure 2B). The pixel diameter and the fluorescence intensity threshold can be easily adapted to fit other cell types and fluorescent proteins. Appropriate values for nuclear diameter and fluorescence intensity need to be determined empirically in any new experimental setting. Further details are given in Supplementary Note 1.

**Figure 3:**
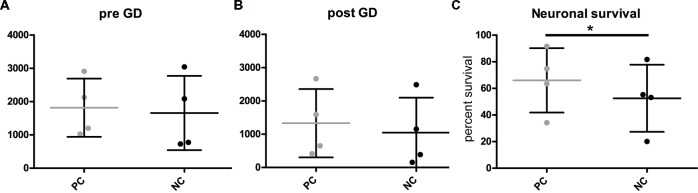
Quantification of neuronal survival after GD.** (A,B)** Cell counts before (pre) and after (post) GD do not readily distinguish the different groups. However, **(C)** calculating the percentage of surviving cells illustrates the capacity of this assay to investigate protective gene function. PC, positive control or NC, negative control. Plots show individual data points from four independent experiments and mean and standard deviation, *p = 0.0091 (two-tailed paired *t*-test).

To investigate gene function in the context of metabolic stress, microscopy of H2B-GFP positive neurons was performed at two time-points: immediately before detrimental glucose deprivation (pre GD) and 24 h after glucose deprivation (post GD). However, neuronal cell numbers in these types of assays are notoriously variable (Figures 3A,B). Thus, simply analyzing the number of nuclei before (Figure 3A) and after GD (Figure 3B, Supplementary Figure S2) of PC and NC is not sufficient to detect differences between the groups. We propose calculating the percentage of surviving cells (i.e., identified “live” nuclei) after GD by dividing the number of counted nuclei after treatment by the number of nuclei detected before treatment. This provides a measure for the relative comparison of anti- or pro-apoptotic effect of any gene of interest compared to the respective controls. As expected (Mergenthaler et al., 2012), Bcl-XL (PC) protected neurons from GD compared to the NC, thereby demonstrating the anti-apoptotic function of this gene (Figure 3C). Consequently, these results demonstrate that this assay is suitable to investigate the function of an overexpressed gene of interest on neuronal survival after stress.

## Discussion—Advantages and Limitations

We demonstrated that the assay described herein functions as a versatile tool to gain important insights into the functional effects of a gene of interest on neuronal survival after metabolic stress (i.e., GD). While many methods to measure cell survival or cell death parameters exist (Galluzzi et al., 2009), biochemical population-based methods are still commonly used to investigate these processes (Riss et al., 2004). A drawback of biochemical methods is that different cell types present in one cell population cannot be distinguished. As a result, measured effects often are generalized, and detailed information on cell population composition are missing.

In contrast, automated image analysis can measure features of single cells, rather than producing a score for the entire image or cell population. The reaction of individual cells to a given stimulus can vary to a large extent. For example, multiparametric measurements of single cells have proven to be advantageous over whole-population measurements (e.g., western blot) for deriving causal protein-signal networks (Sachs et al., 2005) or for protein localization classification (Chen and Murphy, 2005) and screening for toxic compounds (Collins et al., 2015). By using cell-type specific promoters to overexpress the nuclear marker (i.e., H2B-GFP) and the gene of interest only in one cell species, the described assay provides the opportunity to measure effects of exactly one cell-type. Similar results could be obtained using cell-type specific transcription factors as fluorescence markers (i.e., immunofluorescence staining). In addition to the overexpression approach we used in this study, the same approach could be used to express short hairpin (sh)RNAs targeting the gene of interest in conjunction with the fluorescent protein. Likewise, we expect that the image processing approach described herein will also work with common live cell stains such as Hoechst 33342, Draq5, or the recently described live cell far-red nuclear dye SiR-Hoechst (Lukinavičius et al., 2015). Such an approach would be useful when large cell populations rather than (transfected) sub-populations need to be investigated, such as in live cell compound screening. Given the different spectral properties of these dyes, thresholding parameters may need to be adjusted empirically. In principle, our approach may be expanded to incorporate additional cellular markers such as mitochondria. Indeed, mitochondria play an important role in neuronal stress response (Dirnagl and Meisel, 2008) and a large variety of probes and biosensors have been developed to investigate mitochondrial function.

Our approach is simple on purpose as it can be rapidly adapted even by novice users. Even though the relatively simple task of manually counting fluorescent neuronal nuclei is feasible in principle (Supplementary Figure S3, Supplementary Table S1), performing image cytometry circumvents this tedious and error-prone task. Furthermore, image cytometry performs this task with higher fidelity and even large scale experiments can be quantified in a short period of time (Carpenter et al., 2006). Indeed, using our approach thousands of cells can easily be analyzed in a short time. Furthermore, CellProfiler has been used to create elaborate image processing pipelines, such as to differentiate apoptosis and necrosis after excitotoxicity in cerebellar granule neurons (Anilkumar et al., 2017). However, our approach may be limited when used in a complex biological context or to detect rare subpopulations. Modern microscopes can take thousands of images with excellent resolution in the setting of high content screens or time-lapse experiments employing RNA interference (RNAi), chemical libraries or expression plasmids (Carpenter and Sabatini, 2004; Moffat et al., 2006). In such a context, only advanced automated image analysis may yield meaningful results, as important image features may not be readily known. Likewise, our approach is limited to two-dimensional microscopy. The advent of human brain organoid models (Paşca, 2018) as well as 2-photon or light sheet microscopy of live rodent brains, brain slices or whole drosophila brains require three-dimensional microscopy and 3D image analysis. For this, specialized tools to facilitate single neuron tracing in 3D (Peng et al., 2010) and to normalize fluctuating signal intensities in large 3D image stacks (Yayon et al., 2018) have been developed.

In summary, using the microscopy-based neuronal survival assay described here in combination with automated image analysis employing the open source tool CellProfiler, it is possible to investigate protective or detrimental effects of a specific gene of interest on neuronal survival after functional stimulation such as nutrient starvation. Furthermore, this method can easily be adapted to different cell types, cell markers and different stressors or treatments, thereby making it a powerful and versatile tool for the broad neuroscience community.

## Author Contributions

KW conceived and performed the neuronal survival assay experiments, developed the CellProfiler pipeline, analyzed the data and wrote the manuscript. AM discussed the data and allocated funding support to the project. PM supervised all aspects of this work, analyzed and discussed the data and edited the manuscript. All authors have read and approved the final version of the manuscript.

## Conflict of Interest Statement

The authors declare that the research was conducted in the absence of any commercial or financial relationships that could be construed as a potential conflict of interest.

## Footnotes

^1^http://cellprofiler.org/
