# Supplementary material for: Investigating Gene Function for Neuronal Survival After Metabolic Stress Using Semi-Automated Fluorescence Microscopy and Automated Image Analysis
Source: Front Mol Neurosci. 2018 Nov 2;11:393. doi: 10.3389/fnmol.2018.00393 (PMC6224347; doi:10.3389/fnmol.2018.00393)
Supplement: Supplementary file 1 [file Data_Sheet_1.PDF]

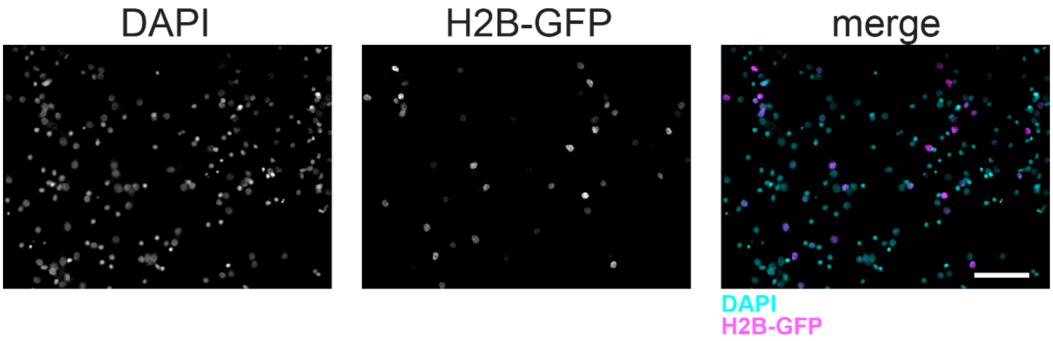

Supplementary Figure 1: Low magnification micrographs of H2B-GFP transfected neurons to provide an overview of culture density and transfection rate. *Scale bar: 100 µm.*

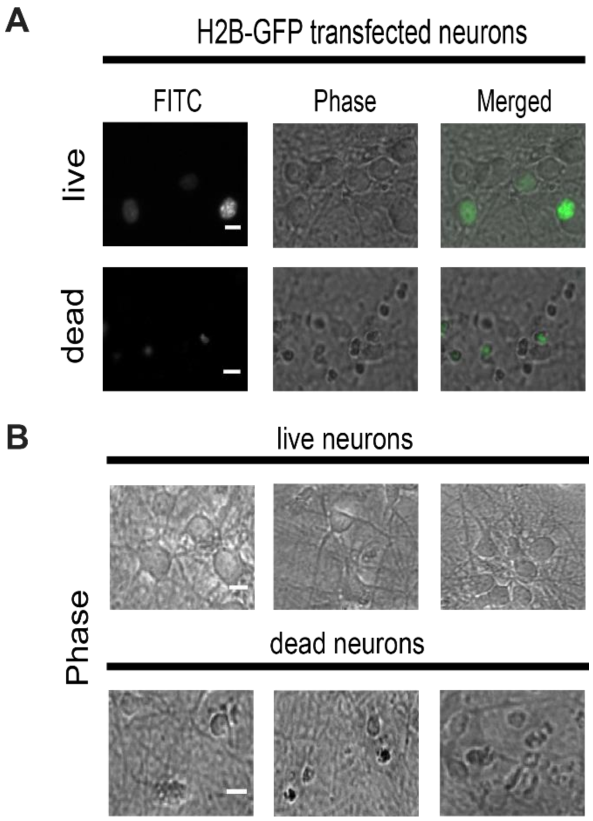

Supplementary Figure 2: H2B-GFP-transfected (**A**, nuclear GFP) and untransfected (**B**) neurons before (live) and after (dead) metabolic deprivation. The images illustrate that both transfected and untransfected neurons are equally susceptible to death after metabolic deprivation. *Phase: phase contrast; scale bars: 10 µm.*

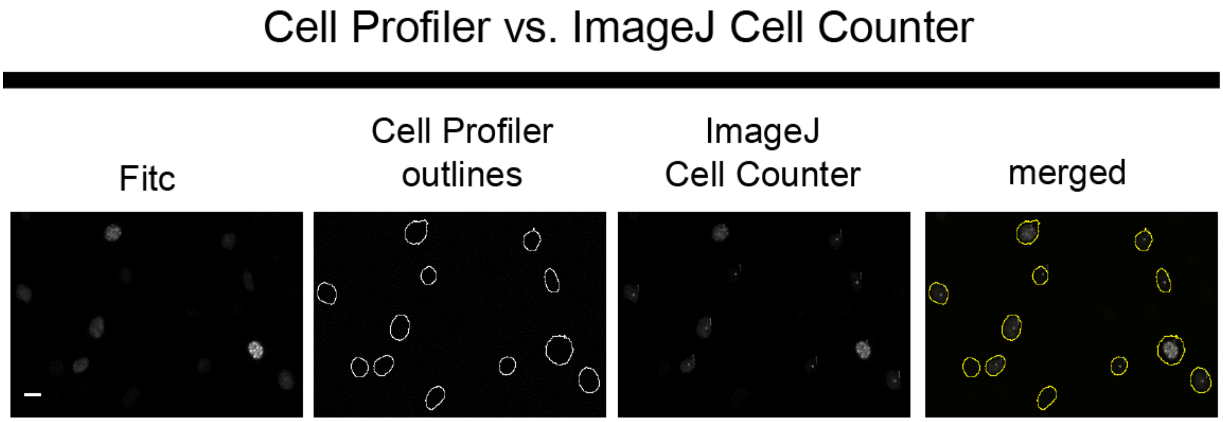

Supplementary Figure 3

Example of CellProfiler counting and manual counting using ImageJ Cell Counter. Shown here is an excerpt of an image captured with a 20x lens. *Scale bar 10 µm.*

Table 1: Nuclei count - CellProfiler versus ImageJ Cell Counter.

Quantitative information about nuclei number counted automatically with the CellProfiler pipeline described herein or counted manually employing the ImageJ Cell Counter tool. For method verification purposes, fluorescent nuclei of one randomly chosen well (16 images) where counted before and after GD resulting in similar nuclei counts (pre GD difference of 105 nuclei counts (i.e. 3.8% difference), post GD difference of 125 nuclei counts (i.e. 5.4%)).

Pre GD	***counted nuclei***		
Image ID	***Cell Profiler***	***ImageJ***	***+/-***
497	149	127	22
498	109	107	2
499	164	152	12
500	161	144	17
501	182	170	12
502	211	200	11
503	148	137	11
504	138	143	-5
505	180	169	11
506	176	169	7
507	169	163	6
508	212	192	20
509	185	204	-19
510	203	207	-4
511	196	189	7
512	164	169	-5
sum	***2747***	***2642***	***105***

Post GD	***counted nuclei***		
Image ID	***Cell Profiler***	***ImageJ***	***+/-***
497	114	98	16
498	100	106	-6
499	161	148	13
500	139	128	11
501	155	160	-5
502	164	158	6
503	135	107	28
504	139	129	10
505	122	110	12
506	149	147	2
507	157	157	0
508	159	154	5
509	160	153	7
510	171	154	17
511	161	156	5
512	152	148	4
sum	***2338***	***2213***	***125***

Supplementary Note 1: Outlined below is the workflow and the individual modules of the Cell Profiler pipeline

Input modules:

Images: Open or drop files and folders here to create a list of all images. It is not necessary to sort pictures within the list.

Names and types: Selection of images → For example: 3 Channel images → DAPI, FITC and TRITC. To analyze only one channel or to analyze images from each channel differently chose images from the list based on regular expressions in the image file name. Here, we only used one channel (nuclei), which was selected with a "ch00" file name suffix.

Analysis modules:

Identify primary objects: This module identifies H2B-GFP positive neuronal nuclei based on following parameters:

Typical diameter (for neurons in this study)	23 – 40 pixels
Threshold strategy	per object
Thresholding method	Mixture of Gaussian (MoG)
Approximate fraction of image covered with objects	0.1
Threshold correction factor	1
Lower and upper bounds on threshold	0.0003 – 0.5 pre GD<br>0.0009 – 0.04 post GD
Method to distinguish clumped objects	Intensity

Measure object intensity: Lists intensities of detected nuclei. Control tool to empirically determine lower and upper bounds on threshold. Furthermore, analysis of different groups of nuclei based on fluorescence intensity could be performed.

Save images: As a control step, images displaying detected and quantified nuclei as outlines are saved. This step is helpful to adapt the pipeline to other cell types. After running the pipeline with a few representative images, outlined images and input images can easily be compared with regards to the accuracy of nuclear counts. If the accuracy of detection is not sufficiently high, adjustments on pixel diameter or threshold boundaries may be advisable.

Export to spreadsheet: Outputs the detected nuclei number per image as a csv file. Additionally, all measured features will be listed. This module allows easy accessibility of the collected data and facilitates further analysis.
